# NiFe_2_O_4_@ nitrogen-doped carbon hollow spheres with highly efficient and recyclable adsorption of tetracycline†

**DOI:** 10.1039/c9ra00670b

**Published:** 2019-04-03

**Authors:** Zhe Chen, Dongzhao Mu, Feng Chen, Naidi Tan

**Affiliations:** School of Material Science and Technology, Jilin Institute of Chemical Technology Jilin 132022 PR China chenzhecz999@163.com; Jinlin Petrochemical Company Organic Synthetic Plants Jinlin 132021 P. R. China

## Abstract

Antibiotics can affect ecosystems and threaten human health; therefore, methods for removing antibiotics have become a popular subject in environmental management and for the protection of human health. Adsorption is considered an effective approach for the removal of antibiotics from water. In this study, NiFe_2_O_4_@nitrogen-doped carbon hollow spheres (NiFe_2_O_4_/NCHS) were synthesized *via* a facile hydrothermal method followed by calcination using NCHS as a hard template. The nanocomposite exhibited high adsorption activity and good recyclability. The nanocomposite was characterized by X-ray diffraction, field emission scanning electron microscopy, transmission electron microscopy, X-ray photoelectron spectroscopy, and nitrogen adsorption–desorption to study its micromorphology, structure, and chemical composition/states. In addition, the factors affecting the adsorption process were systematically investigated, including tetracycline (TC) concentration, solution pH, ionic strength, and temperature. The maximum adsorption capacity for TC was calculated to be 271.739 mg g^−1^ based on the Langmuir adsorption model, which was higher than various other materials. This study provides an effective method for constructing the NiFe_2_O_4_/NHCS core–shell structure, which can be applied for the removal of TC from water.

## Abstract 2

Antibiotics can affect ecosystems and threaten human health; therefore, methods for removing antibiotics have become a popular subject in environmental management and for the protection of human health.

## Graphical abstract

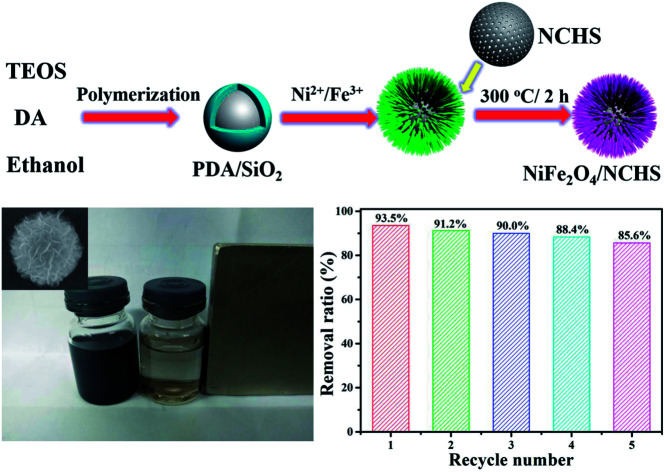

## Introduction

Antibiotics have been one of the greatest discoveries for humans, and various types of antibiotics have been excessively used in human and veterinary medicine, and in aquaculture.^1–3^ However, the widespread use of antibiotics and the large amounts excreted by humans and animals have resulted in increasingly more pharmaceuticals being discharged into wastewaters and manures, which have led to adverse effects on ecosystem health.^4,5^ Therefore, the development of efficient methods to effectively eliminate antibiotics from wastewater is an important research topic for human health and environmental system management.

To date, many researchers have developed effective methods to remove traditional pollutants from the aquatic environment, such as adsorption,^5,6^ coagulation/flocculation,^7^ photocatalysis,^8–10^ chemical oxidation,^11^ ion-exchange,^12^ biodegradation,^13^ and advanced oxidation.^14^ Of these, adsorption is the most pragmatic and convenient method for the removal of antibiotics from water sources, owing to its easy operation, convenience, low energy requirements, high efficiency, cost effectiveness, and facile recovery or reuse of the adsorbent. In addition, this process does not produce secondary pollutants. Hence, the selection of an appropriate adsorbent is an important and imminent task for the management of the aquatic environment. Until now, activated carbon,^15^ metal oxides,^16^ clay minerals,^17^ and silicon nanomaterials^18,19^ have been applied as conventional adsorbents for the removal of antibiotics from wastewater. However, the low adsorption capacity, high cost, and poor recyclability have restricted their practical application. Thus, the application of magnetic adsorbents is an innovative method to resolve environmental problems.^20–22^ For example, multifunctional magnetic microspheres (Mag@ZnO–Co_3_O_4_) with bimetal oxide shells have been synthesized and showed excellent adsorption properties for oxytetracycline, with the maximum adsorption capacity of oxytetracycline found to be 128 mg g^−1^,^23^ and core–shell Fe_3_O_4_@ZIF-8 has been prepared and showed excellent adsorptive capacity for tetracycline (TC) antibiotics.^24^ However, there is still a challenge in producing a new generation of separation media magnetic nanocomposites.

In recent years, transition-metal oxides such as ZnFe_2_O_4_, MnFe_2_O_4_, and NiFe_2_O_4_ have attracted extensive attention as anode materials,^25–27^ photocatalyst materials,^28,29^ electronics materials,^30^ and solar cell materials^31^ because of their low cost, excellent visible light response, and good photochemical stability. However, seldomly have these transition-metal oxides been used as adsorption materials.

To the best of our knowledge, this is the first study to fabricate and grow magnetic porous NiFe_2_O_4_ nanosheets on N-doped carbon hollow spheres (NCHS), where NCHS also served as a hard template, which utilized a facile, mild, and ecofriendly method. A range of techniques including X-ray diffraction (XRD), scanning electron microscopy/transmission electron microscopy (SEM/TEM), X-ray photoelectron spectroscopy (XPS), nitrogen sorption, and magnetic hysteresis loop were employed to fully characterize the obtained materials. It was found that NiFe_2_O_4_/NCHS had a higher adsorption capacity for TC (271.739 mg g^−1^) than that of pure NiFe_2_O_4_, as well as good recyclability and stability. The results from the present study provide a practical method for the removal of TCs from water. Furthermore, this magnetic material may have potential practical uses in sensors and for energy storage.

## Experimental section

### 1. Preparation of NCHS carbon hollow spheres

All reagents used in the present study were purchased without any further purification. The NCHS was fabricated based on a previously reported method.^32^ Briefly, 12 mL of anhydrous ethanol and 0.5 mL of ammonia (25%) were added to 40 mL of distilled water and vigorously stirred for 20 min, and then 1 mL of TEOS was added dropwise to the above solution. Then, 8 mL of dopamine hydrochloride (DA) aqueous solution (50 mg mL^−1^) was added into the above mixture and the mixture was stirred continuously for 32 h. The material (PDA/SiO_2_) was collected after centrifugation, washed four times with purified water, and then collected and dried *via* vacuum freeze drying. The precipitates were heated at 800 °C for 3 h at a heating rate of 5 °C min^−1^ under a N_2_ atmosphere, and the NCHS was obtained.

### 2. Preparation of NiFe_2_O_4_/NCHS composite

The obtained NCHS (20 mg) was dispersed into 60 mL of deionized water under sonication for 30 min. Then, 0.2 mmol Ni(NO_3_)_2_·6H_2_O, 0.4 mmol Fe(NO_3_)_3_·9H_2_O, 0.38 mmol sodium citrate, 1 mmol NH_4_F, and 5 mmol urea were slowly added into the above suspension solution while being stirred for 60 min. Subsequently, the uniform mixture was transferred to an 80 mL Teflon-lined stainless-steel autoclave and heated at 150 °C for 36 h. The product was then collected after centrifugation and washing several times with distilled water and ethanol. Finally, the obtained product was annealed at 300 °C for 2 h in a N_2_ atmosphere with a heating rate of 2 °C min^−1^. For comparison, NiFe_2_O_4_ was obtained under the same conditions except for the exclusion of NCHS.

## Results and discussion

**Scheme 1:**
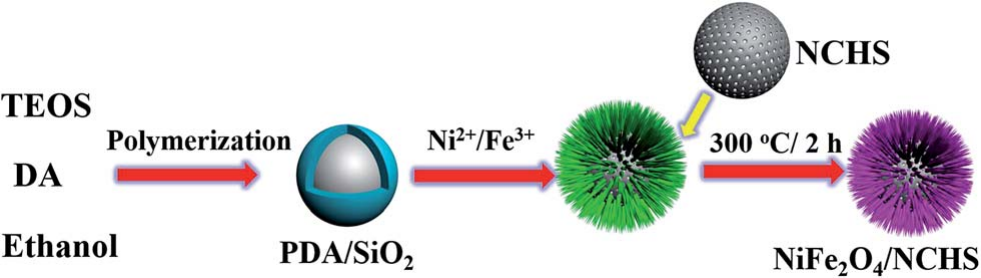
Schematic of the synthesis and application of the NiFe_2_O_4_/NCHS composite.

The synthetic procedure of NiFe_2_O_4_/NCHS is rationally demonstrated in Scheme 1. First, PDA/SiO_2_ with uniform spheres were prepared *via* the hydrolysis of TEOS and the polymerization of dopamine in a mixture of water and ethanol, combined in a one-pot process. Second, Ni^2+^ and Fe^3+^ ions were randomly adsorbed onto the surface of the PDA/SiO_2_ and formed a Ni/Fe-based precursor with a hollow interior under facile hydrothermal treatment in an alkaline solution. Lastly, the NiFe_2_O_4_/NCHS with a hollow structure was obtained after calcination treatment at 350 °C for 2 h in a N_2_ atmosphere.

**Fig. 1:**
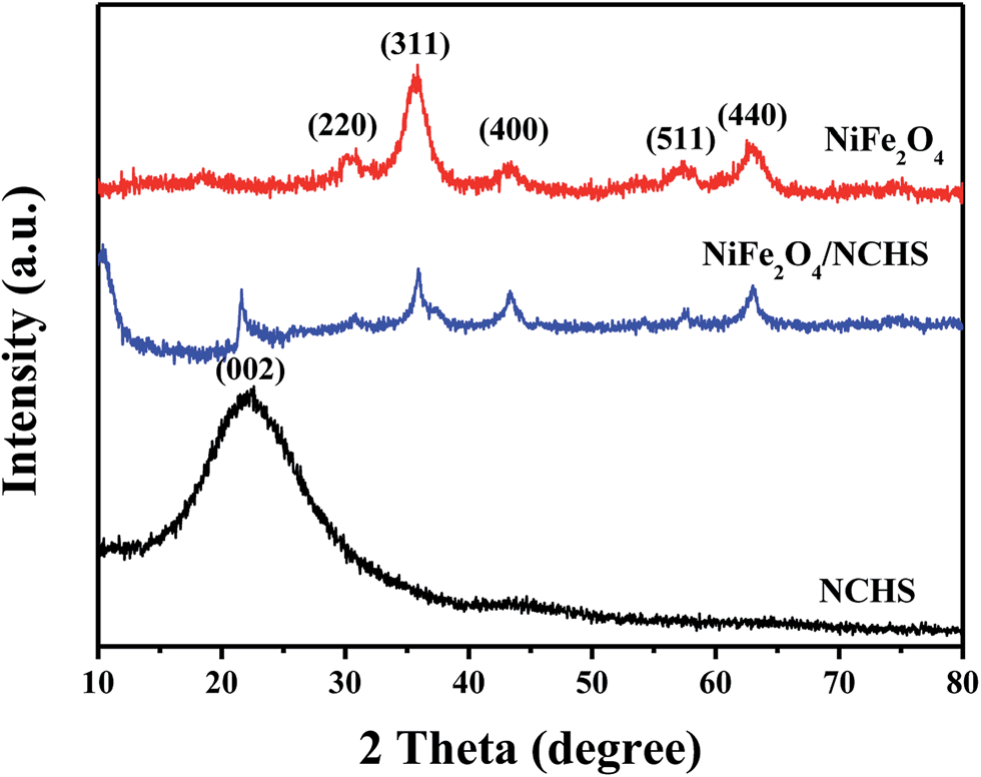
XRD spectra of NiFe_2_O_4_, NiFe_2_O_4_/NCHS and NCHS.

The crystalline structures of the synthesized samples were investigated with XRD, as shown in Fig. 1. The XRD pattern of NiFe_2_O_4_/NCHS displayed broader diffraction peaks of (002) corresponding to graphitized carbon.^33^ In addition, the other characteristic peaks showed the reflection planes of (220), (311), and (400) that were in agreement with the cubic spinel phase of NiFe_2_O_4_ (JCPDS 54-0964).^34^ The peaks were broader than that of pure NiFe_2_O_4_ because of an overlap with the corresponding peak of the NHCS substrate.

**Fig. 2:**
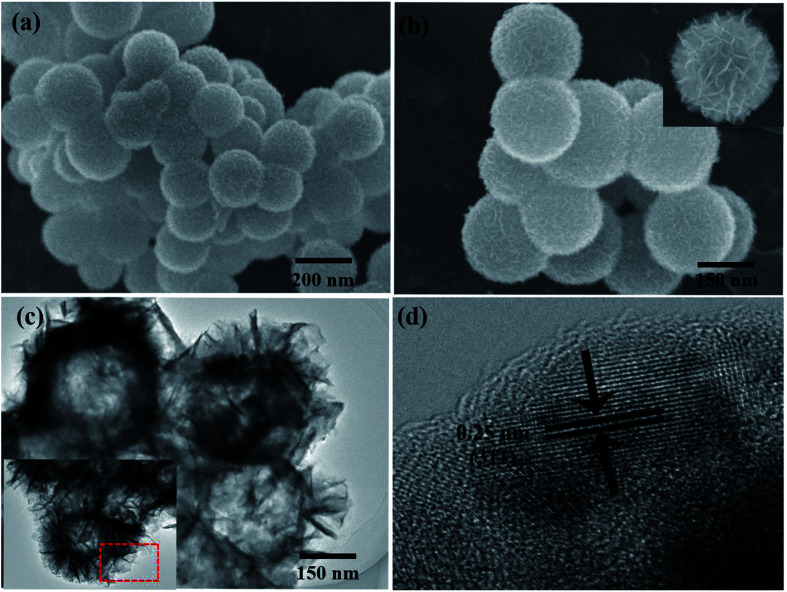
SEM and TEM image of NiFe_2_O_4_/NCHS hollow nanospheres.

Fig. 2a and b show representative field emission SEM (FE-SEM) images of NiFe_2_O_4_/NCHS with hollow nanospheres. Clearly, the sample displays uniformly dispersed spherical structures with an average diameter of approximately 200–300 nm. Moreover, the high-magnification FE-SEM image revealed the sample was composed of nanosheets. The microstructures of the NiFe_2_O_4_/NCHS sample were further characterized using TEM. The NiFe_2_O_4_/NCHS sample exhibited a hollow structure that was composed of interconnected nanosheets, which was similar to the results obtained from the FE-SEM image (Fig. 2c). The resolved interplanar distances of the lattice fringes were 0.25 nm apart, which corresponded to the (311) plane of the face-centered cubic lattice of NiFe_2_O_4_ (JCPDS 54-0964).^35,36^ Importantly, it was found that the pattern and the surface morphology of the adsorbent did not change significantly and remained relatively stable after adsorption (Fig. S2†).

**Fig. 3:**
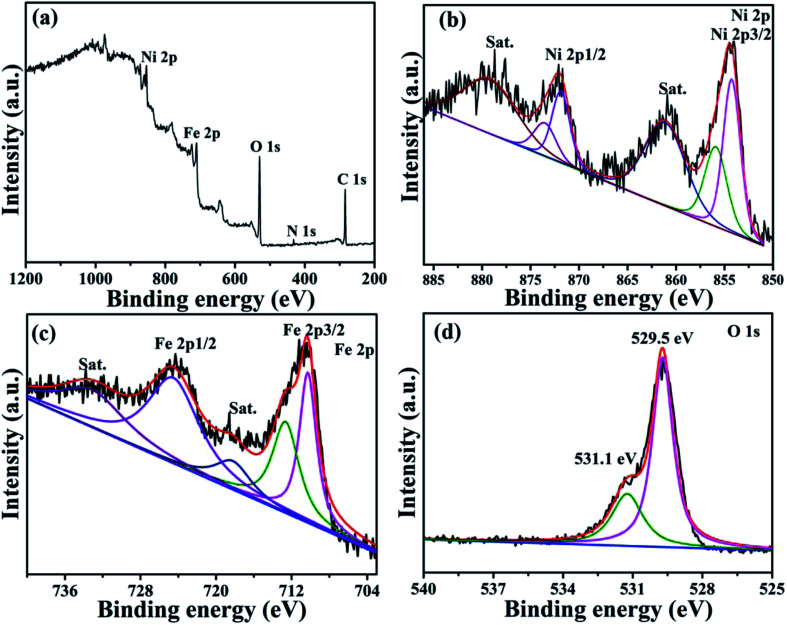
X-ray photoelectron spectroscopy (XPS) spectra of (a) the survey spectrum, (b) Ni 2p, (c) Fe 2p, (d) O 1s for NiFe_2_O_4_/NCHS composite.

The surface chemical composition and structure of the NiFe_2_O_4_/NCHS were further studied with the XPS spectra. The XPS results displayed the existence of Ni, Fe, N, and O elements in the sample (Fig. 3a). For the Ni 2p spectrum shown in Fig. 3b, two main peaks were located at 854.2 and 871.9 eV, corresponding to the two spin–orbits Ni 2p3/2 and Ni 2p1/2, respectively, whereas the binding energy at 718.2 and 724.1 eV were in agreement with Fe 2p3/2 and Fe 2p1/2, respectively. In addition, the two spin–orbits Ni 2p and Fe 2p spectra of NiFe_2_O_4_/NCHS corresponded to two shake-up satellites (denoted as Sat.).^37^ Meanwhile, the spin–orbit peaks in Ni 2p and Fe 2p could be fitted to two distinct peaks, which were attributed to the coexistence of Ni^2+^/Ni^3+^ and Fe^2+^/Fe^3+^ cations, respectively. The O 1s XPS spectrum showed two peaks at binding energies of 529.5 and 531.1 eV, which were consistent with the Ni–Fe–O bonds and oxygenic functional groups.^38^ XPS measurement further revealed the valence state of NiFe_2_O_4_/NCHS after adsorption (Fig. S3†). For comparison, the survey XPS curves of NiFe_2_O_4_/NCHS showed that existence of C, N, and O elements in the sample were remarkable enhancement. Importantly, the Ni 2p spectra of NiFe_2_O_4_/NCHS shifted from 854.2 and 871.9 eV to 856.1 and 873.9 eV, while the Fe 2p spectrum of NiFe_2_O_4_/NCHS shifted from 718.2 and 724.1 eV to 711.4 and 724.7 eV, respectively. This is due to the fact that the hydroxyl, carbonyl and amino groups of the tetracycline molecule provided a large number of electrons to Ni and Fe to form a metal complex. Furthermore, due to forming active oxygen-containing functional groups lead to the positions of O 1s offset occurs. As a result, the tetracycline molecule was successfully adsorbed NiFe_2_O_4_/NCHS.

**Fig. 4:**
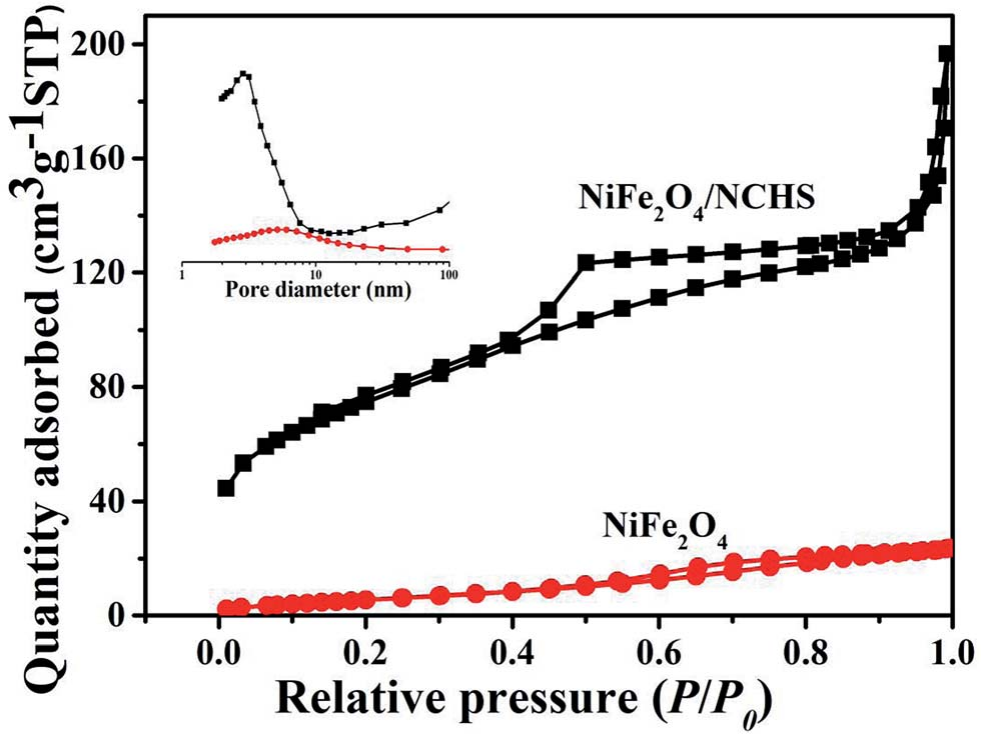
N_2_ adsorption/desorption isotherm and the corresponding pore size distribution (inset) of pure NiFe_2_O_4_ and NiFe_2_O_4_/NCHS composite.

N_2_ adsorption–desorption and pore size distribution were used to analyze the textural properties of the NiFe_2_O_4_ and NiFe_2_O_4_/NCHS composite. As shown in Fig. 4, the N_2_ adsorption–desorption isotherms of the pure NiFe_2_O_4_ and NiFe_2_O_4_/NCHS composite exhibited a type IV isotherm with a type H3 hysteresis loop, which were characteristic of a mesoporous structure.^39^ The hysteresis loop of the NiFe_2_O_4_/NCHS in the broad *P*/*P*_0_ range of 0.45–1 indicated the presence of large mesopores and macrospores, which further confirmed the hierarchically porous structure of this carbon material. The Brunauer–Emmett–Teller specific surface area and the pore volume of NiFe_2_O_4_/NCHS were 268.8 m^2^ g^−1^ and 0.22 cm^3^ g^−1^, respectively, which were much larger than that of pure NiFe_2_O_4_ (21.85 m^2^ g^−1^ and 0.04 cm^3^ g^−1^, respectively), as shown in Table S1.†^40,41^ The increased surface area and hierarchical pore could be attributed to the adsorption of TC. In addition, the pore diameter of NiFe_2_O_4_/NCHS after adsorption of tetracycline was smaller than that of before adsorption, indicating that the pollutants with large molecules may cause blockage of the external channel of adsorbent pores with small diameters (Fig. S4†). Zeta potential measurements were carried out at pH 7 to investigate the surface charge of the samples in aqueous solution. As displayed in Fig. S5,† the TC dispersions are positive charged with a zeta potential of about 36.31 mV. Upon addition of NCHS, the porous carbon shell can immediately adsorb Ni^2+^ and Fe^3+^ from the solution, resulting in the crystal nucleation and formation of Ni(OH)_2_ and Fe(OH)_3_ on the surface of porous NCHS. These initial Ni(OH)_2_ and Fe(OH)_3_ precipitates on the surface of NCHS could provide nucleation spots to direct the growth of Ni(OH)_2_ and Fe(OH)_3_ nanosheets. Finally, loose Ni(OH)_2_ and Fe(OH)_3_ nanosheets were vertically grown on the surface of NCHS by self-assembly. After calcination at 300 °C for 2 h in O_2_, nanoflower-like Ni(OH)_2_/Fe(OH)_3_/NCHS composite were transformed to NiFe_2_O_4_/NCHS. The NiFe_2_O_4_/NCHS possess a negative ζ potential of −20.16 mV. The good dispersity and stability of NiFe_2_O_4_/NCHS/TC in the suspension, this indicates that tetracycline hydrochloride is not easy to be separated from NiFe_2_O_4_/NCHS suspension and can achieve good adsorption effect.

**Fig. 5:**
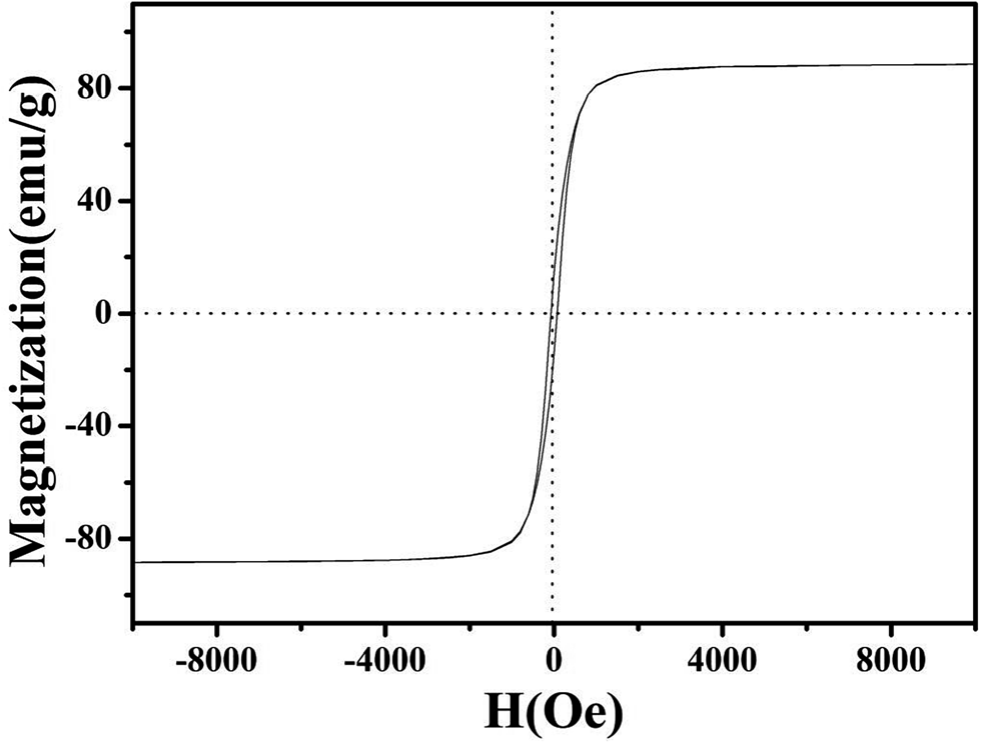
Magnetic hysteresis loop of NiFe_2_O_4_/NCHS.

The magnetic properties of NiFe_2_O_4_/NCHS were further analyzed at room temperature (25–30 °C), as shown in Fig. 5. The field-dependent magnetization curves were completely reversible, which further indicated that the as-synthesis materials were superparamagnetic without coercivity and remanence. The saturation magnetization value of 85 emu g^−1^ demonstrated its excellent magnetic properties, allowing it to be easily separated from the treated samples by an external magnet.^42^

**Fig. 6:**
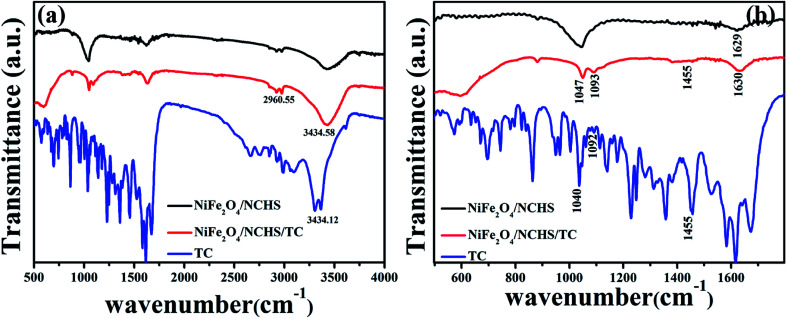
FT-IR spectra of NiFe_2_O_4_/NCHS before adsorption and after adsorption of tetracycline.

In order to examine the surface groups of NiFe_2_O_4_/NCHS before and after adsorbent, the FTIR spectra were performed (Fig. 6). The absorption peaks in the range from 1700–1200 cm^−1^ is attributed to the skeleton vibration absorption peaks of the benzene ring in the tetracycline molecule.^43^ The characteristic peak of NiFe_2_O_4_/NCHS/TC at 1455 cm^−1^ represent the skeleton vibration of the benzene ring in the tetracycline molecule. The absorption peak at 1040 cm^−1^ is ascribed to the stretching vibration of C–OH of the tetracycline molecule, which was moved to the 1047 cm^−1^ after tetracycline adsorption due to the tetracycline molecule was participated in its adsorption through hydrogen bond. Based on the above analysis, the tetracycline molecule was successfully adsorbed NiFe_2_O_4_/NCHS.

**Fig. 7:**
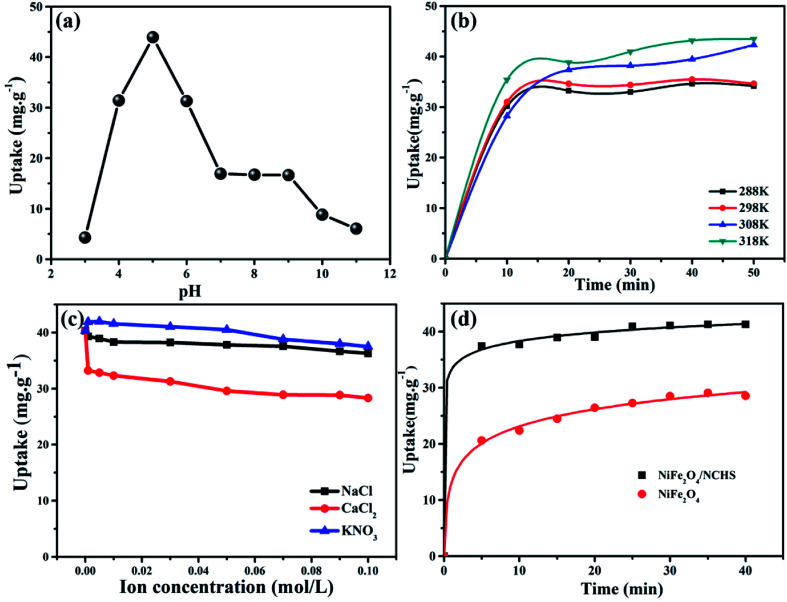
Effect of (a) pH, (b) temperature, (c) ion species (d) different sample of absorption amount over time on TC adsorption (*T* = 298 K; initial TC concentration = 10 mg L^−1^; adsorbent dose = 10 mg L^−1^; pH = 5).

The adsorption behavior of TC using NiFe_2_O_4_/NCHS was influenced by pH ranging from 3.0 to 11.0 with an initial concentration of 10 mg L^−1^ at 298 K (Fig. 7a).^43–46^ The adsorption of TC was increased with increasing pH from 3.0 to 5.0, and the maximum adsorption capacity for TC was reached at 43.95 mg g^−1^ when the solution pH was 5. This was attributed to electrostatic and complexation attractions; thus, increasingly more TC was easily attracted to the surface of NiFe_2_O_4_/NCHS. However, the adsorption capacity of TC showed a similar rapidly decreasing uptake trend when pH values ranged from 5 to 7. Subsequently, when pH values exceeded 9, no significant effect was found except for a slight decrease. Therefore, the optimum pH is a highly significant factor for controlling the adsorption capacity of TC.

To investigate the effect of temperature on the adsorption process, a batch experiment was undertaken at four different temperatures (Fig. 7b) with an initial concentration of 10 mg L^−1^ at a pH of 5. The adsorption performance of the NiFe_2_O_4_/NCHS sample was significantly improved with an increase in temperature from 288 K to 318 K, causing the adsorption amount to quickly increase after 50 min from 32.5 mg g^−1^ to 41.3 mg g^−1^. Hence, the adsorption process of TC on the NiFe_2_O_4_/NCHS sample was an endothermic process based on the calculated values of Gibb's free energy.

The removal of pollutants is often influenced by salt (especially inorganic salt) or ions (*e.g.*, Na^+^, Ca^2+^, K^+^, Mg^2+^, Al^3+^, and so on) in water and further affects the adsorption capacity of TC. Three commonly encountered inorganic salts, NaCl, CaCl_2_, and KNO_3,_ in tap water and natural water were selected to investigate the effect of coexisting ionic species on the adsorption of TC (Fig. 7c). The different concentrations of NaCl and KNO_3_ had little effect on the adsorption of TC. By contrast, after the addition of CaCl_2_ the adsorption capacity of TC was obviously decreased, which implies that it competes with TC for the active site on the surface of the adsorbent, causing a rapid decrease in the adsorption process of TC.^47,48^

The kinetic curves for TC adsorption of NiFe_2_O_4_/NCHS and NiFe_2_O_4_ are shown in Fig. 7d. The two curves revealed that the initial TC uptake rate was quite fast during the first 20 min and reached adsorption equilibria within 40 min. Besides, because the NiFe_2_O_4_/NCHS sample had a much larger specific surface area and more positive surface charge, it showed faster adsorption rates and enhanced adsorption capacity than that of NiFe_2_O_4_ under the same experimental conditions. The maximum TC adsorption quantities for the NiFe_2_O_4_/NCHS and NiFe_2_O_4_ samples were 41.30 and 28.70 mg g^−1^, respectively, after 40 min of adsorption, with NiFe_2_O_4_/NCHS having more adsorption sites and faster adsorption rates than that of NiFe_2_O_4_. In addition, a series of NiFe_2_O_4_/NCHS with different dosages of Ni and Fe, pure NiFe_2_O_4,_ and NCHS were tested as adsorbents for TC adsorption (Fig. S1†). Comparing the different samples, NiFe_2_O_4_/NCHS exhibited much higher adsorption capacity than that of NiFe_2_O_4_ and NCHS. In addition, under a dosage of Ni and Fe of 0.1 : 0.2, NiFe_2_O_4_/NCHS revealed the best adsorption quantity of TC.

**Fig. 8:**
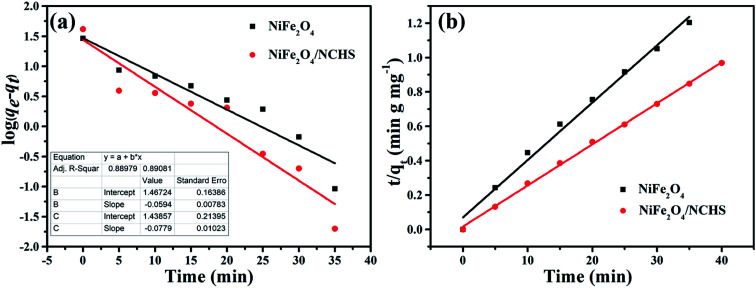
The kinetic models for TC adsorbed on the samples: (a) pseudo-first-order, (b) pseudo-second-order.

Two well-known kinetic models were adopted to analyze the adsorption mechanism of NiFe_2_O_4_/NCHS and pure NiFe_2_O_4_, as shown in Fig. 8. The corresponding parameters (*k*_1_ and *k*_2_) obtained by linear regression are listed in Table S2.† The results showed that all experimental data were distributed on the fitted straight lines, which indicated that it did not match well with the pseudo-first-order model (Fig. 8a).^49,50^ However, the experimental data displayed good agreement with the pseudo-second-order model owing to the calculated *q*_e_ values being matched with the experimental adsorption amount and values of *R*^2^ were higher than 0.995 for NiFe_2_O_4_/NCHS (Fig. 8b). Hence, the pseudo-second-order kinetic model was more appropriate than the pseudo-first-order kinetic model for the adsorption process, which displayed the adsorption process of tetracycline on NiFe_2_O_4_/NCHS was more in accordance with chemical adsorption. In summary, the adsorption process of tetracycline NiFe_2_O_4_/NCHS was based on chemical adsorption, which was supplemented by physical adsorption. This is because tetracycline could be bound to metal ions (Ni^2+^, Fe^3+^) to form an antibiotic–metal complex owing to its electron donor groups which could form strong coordination.

**Fig. 9:**
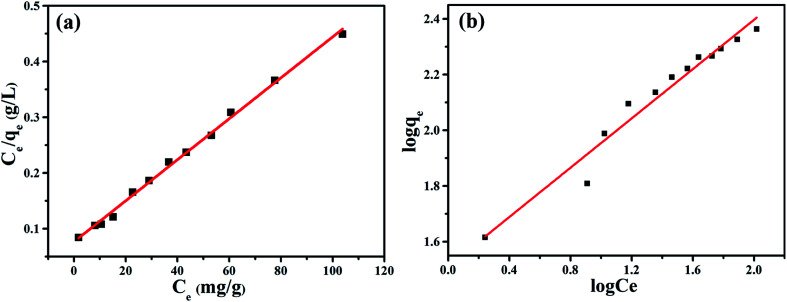
(a) Langmuir and (b) Freundlich modeling of the isotherms for TC on the sample NiFe_2_O_4_/NCHS composite at 30 °C.

The Langmuir and Freundlich isotherm equations were used to analyze the adsorption process of NiFe_2_O_4_/NCHS for TC (Fig. 9).^51–54^ The respective fitted parameters of the Langmuir isotherm equation and Freundlich model are listed in Table S3.† Compared with the different correlation coefficient (*R*^2^) values, the Langmuir model simulation (*R*^2^ ≥ 0.994) was larger than the Freundlich isotherm model (*R*^2^ ≥ 0.960); therefore, the Langmuir adsorption model was more suitable to describe the TC adsorption equilibrium process. The maximum TC adsorption capacity of NiFe_2_O_4_/NCHS based on the Langmuir equation was 271.739 mg g^−1^, which was consistent with the experimental data.^55^ In addition, the maximal TC adsorption capacity of NiFe_2_O_4_/NCHS was superior to that of previously reported materials (Table S4†).

**Fig. 10:**
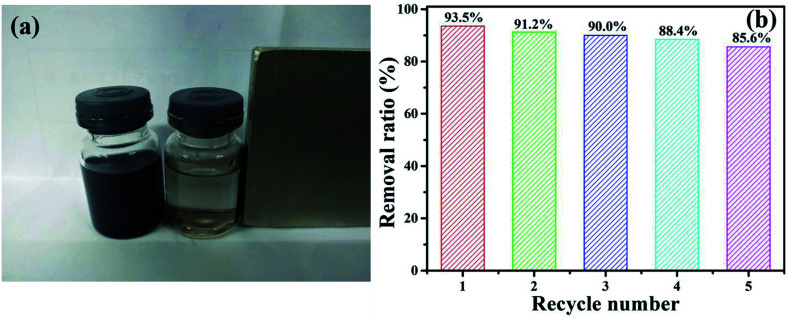
(a) The digital photograph of the NiFe_2_O_4_/NCHS responding to a magnet, and (b) TC removal efficiency of the sample in recycle experiments.

To evaluate the performance of NiFe_2_O_4_/NCHS for the removal of TC, the reusability and stability of the sample were studied (Fig. 10). NiFe_2_O_4_/NCHS was collected from the aqueous suspension by magnetic separation. The experiment results showed that there was a slight adsorbed activity loss of TC at the 4 cycle and 5 cycle, and there was over 85.6% TC removal ratio achieved after 5 cycle tests. In addition, the adsorption–desorption cycle studies were implemented to access the regeneration of NiFe_2_O_4_/NCHS. Adsorption desorption and regeneration deeply studies. The adsorbed NiFe_2_O_4_/NCHS was desorbed by NaOH and NH_3_·H_2_O to achieve the regeneration of NiFe_2_O_4_/NCHS after equilibrium adsorption. The regeneration efficiency of NiFe_2_O_4_/NCHS and removal ratio of TC was illustrated in Fig. S6.† It was clearly observed that although the adsorption capacity of NiFe_2_O_4_/NCHS decreased with the increment in the times of adsorption–desorption of the adsorbent, the removal ratio of tetracycline was maintained at 80% or more after five times of adsorption–desorption cycles of the adsorbent with NaOH(0.001 M)/NH_3_·H_2_O(0.01 M), which indicated that NiFe_2_O_4_/NCHS had a relatively good reusability and were a promising candidate for tetracycline adsorption in the practical condition.^56–62^ Therefore, the as-prepared sample possessed an efficient and stable adsorbed TC activity, making it useful for the removal of TC from water.

## Conclusions

In summary, NiFe_2_O_4_/NCHS was prepared *via* a simple hydrothermal method followed by calcination using NCHS as a hard template. The nanocomposite was characterized by a range of techniques to study its micromorphology, structure, and chemical composition/states. The NiFe_2_O_4_/NCHS had a large specific surface area and good adsorption capacity for TC. Based on the experimental results, we found that the adsorption kinetics followed the pseudo-second-order model and the adsorption isotherms obeyed the Langmuir adsorption model. Importantly, NiFe_2_O_4_/NCHS can be effectively separated for reuse by applying an external magnetic field and it has an efficient and stable adsorbed TC activity, thus making it useful for the practical removal of TC from water. In addition, this magnetic material may have potential practical uses in sensors and for energy storage. The present study provides an effective approach to construct other hierarchical carbon–bimetal oxide composite materials.

## Conflicts of interest

The authors declare no competing financial interest.

## Supplementary Material

RA-009-C9RA00670B-s001
