# Supplementary material for: NiFe2O4@ nitrogen-doped carbon hollow spheres with highly efficient and recyclable adsorption of tetracycline
Source: RSC Adv. 2019 Apr 3;9(19):10445–53. doi: 10.1039/c9ra00670b (PMC9062489; doi:10.1039/c9ra00670b)
Supplement: RA-009-C9RA00670B-s001 [file RA-009-C9RA00670B-s001.pdf]

Electronic Supplementary Material (ESI) for RSC Advances.
This journal is © The Royal Society of Chemistry 2019

Supporting Information for

$NiFe_2O_4$@ Nitrogen-Doped Carbon Hollow Spheres with highly efficient and recyclable adsorption of tetracycline hydrochloride

Zhe chen[a], Dongzhao mu[a], Feng chen[b], and Naidi tan[a*]

[a] *School of Material Science and Technology, Jilin Institute of Chemical Technology, Jilin, 132022, PR China,*

E-mail: chenzhecz999@163.com

[b] *Jinlin Petrochemical Company Organic Synthetic Plants, Jinlin, 132021, P. R. China*

1. Characterizations

A D/Max-RB X-ray diffractometer (Rigaku, Japan) was used to obtain X-ray diffraction (XRD) patterns. TEM images were obtained on a transmission electron microscope (JEM-2100F, JEOL, Japan) with an accelerating voltage of 200 kV. FESEM images were recorded on a field emission scanning electron microscope (JSM-7500, JEOL, Japan) with an accelerating voltage of 15 kV. Chemical states of elements were measured on an X-ray photoelectron spectroscopy (XPS) equipped with an ultra-high vacuum VG ESCALAB 210 electron spectrometer. Fourier transform infrared spectra (FTIR) were measured on an IR Affinity-1 FTIR spectrometer (Shimadzu, Japan). N_2 adsorption-desorption isotherms were measured on a Micromeritics ASAP 3020 equipment (USA). All as-synthesized samples were degassed at 180 °C for 5 h prior to adsorption measurements. Specific surface area was determined by the multipoint Brunauer–Emmett–Teller (BET) method. The magnetization curve was conducted on Quantum Design MPMS-7 SQUID magnetometer at 300 K under varying magnetic field.

2. Adsorption experiment

Table 1. The corresponding physicochemical properties of prepared samples.

Sample	Average crystal size [nm] (standard deviation)	S_{BET} [m^2g^{-1}]	Pore volume [cm^3g^{-1}]
$NiFe_2O_4$	6.46	21.85	0.04
$NiFe_2O_4$/NCHS(before)	5.34	335.9	0.68
$NiFe_2O_4$/NCHS(after)	3.38	268.8	0.22

Table 2. Pseudo-first-order and pseudo-second-order kinetic parameters of the as-prepared samples.

Samples	$q_{e,exp}$ ($mg.g^{-1}$)	Pseudo-first-order model			Pseudo-second-order model		
		$q_{e,cal}$ ($mg.g^{-1}$)	k_1 ($\times 10^{-2} min^{-1}$)	R^2	$q_{e,cal}$ ($mg.g^{-1}$)	K_2 ($\times 10^{-2} g.mg^{-1} min^{-1}$)	R^2
NiFe	29.11	29.33	13.68	0.890	29.96	1.60	0.989
NiFe- NCHS	41.28	27.45	3.39	0.891	41.84	3.44	0.999

Table 3. Parameters of Langmuir isotherm and Freundlich isotherm for TC adsorption on $NiFe_2O_4$/NCHS.

Samples	Langmuir model			Freundlich model		
	q_{max} ($mg.g^{-1}$)	K_L ($L\ mg^{-1}$)	R^2	K_F $(mg/g)(L/mg)^{1/n}$	n	R^2
NiFe- NCHS	271.739	0.048	0.996	32.478	2.258	0.960

Table 4. The maximum adsorption capacity for TC by different adsorbents for comparison

Samples	q_{max} (mg/g)	References
Mag@ZnO-Co_3O_4	128	1
graphene oxide	212	2
GO-MPs	39.1	3
Ni nanoparticles/silica (Ni NPs/SiO_2)	381.3	4
MWCNT	269.54	5
activated sludge	91	6
NHCS-NiFe	271.739	This work

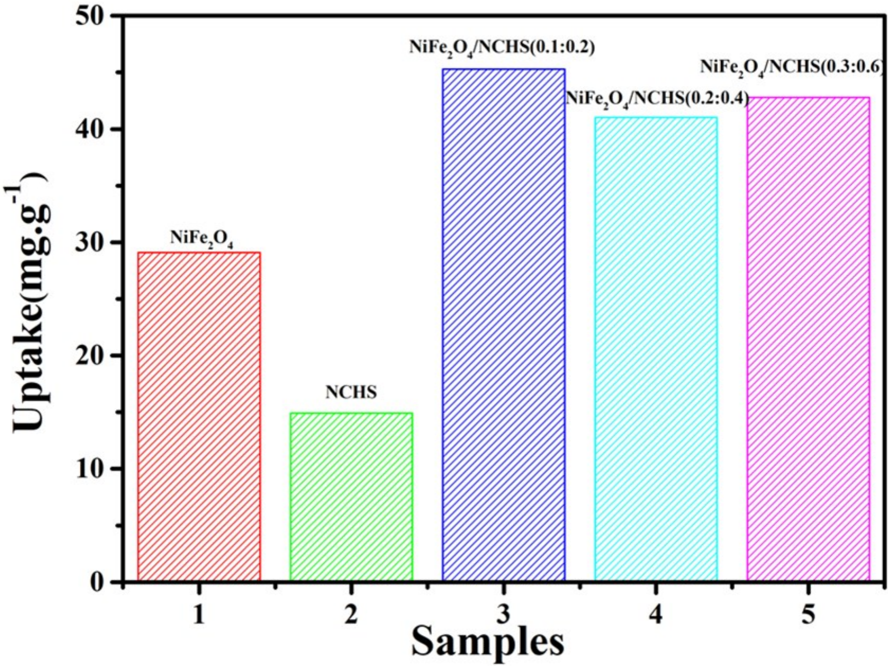

Figure S1. Adsorption of TC by magnetic composites with different concentration of raw materials.

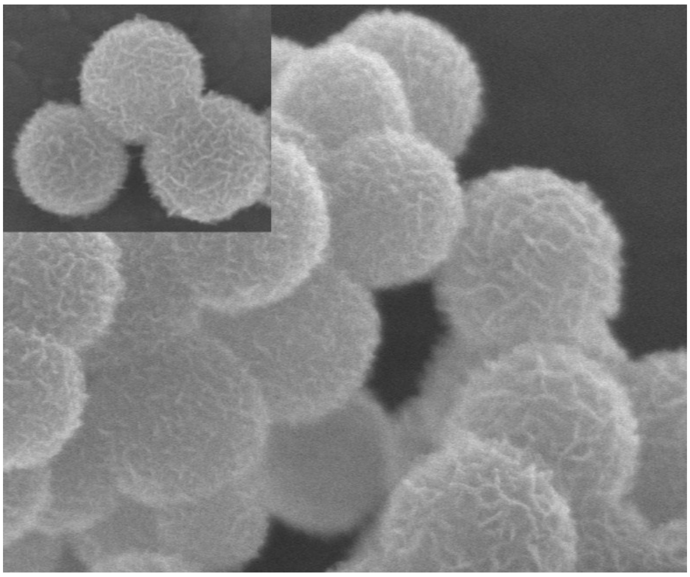

Figure S2. The SEM images of $NiFe_2O_4/C$ samples after absorption.

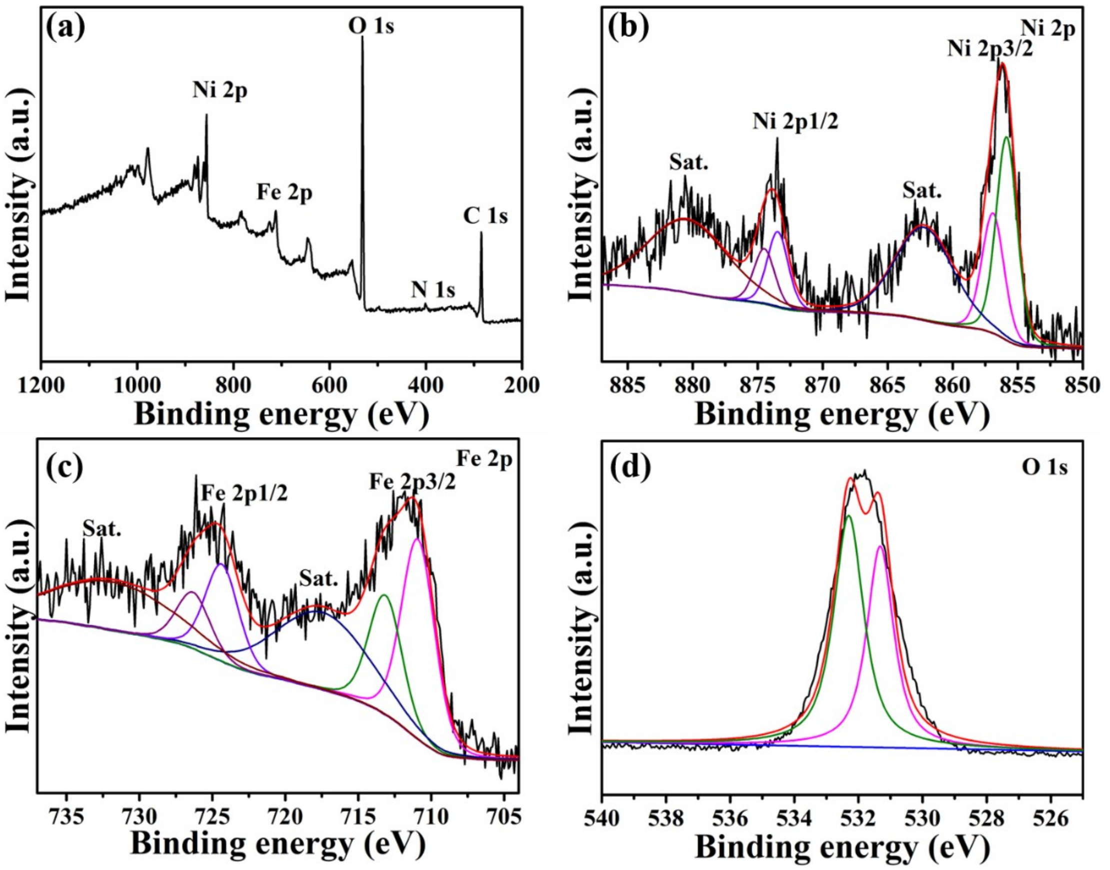

Figure S3. X-ray photoelectron spectroscopy (XPS) spectra of (a) the survey spectrum, (b) Ni 2p, (c) Fe 2p, (d) O 1s for $NiFe_2O_4$/NCHS composite after adsorption.

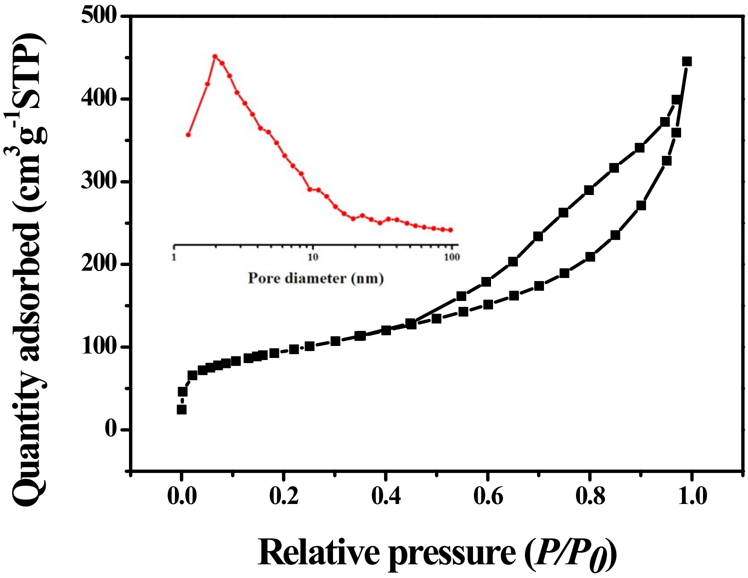

Figure S4. N_2 adsorption/desorption isotherm and the corresponding pore size distribution (inset) of the adsorbed $NiFe_2O_4$/NCHS composite.

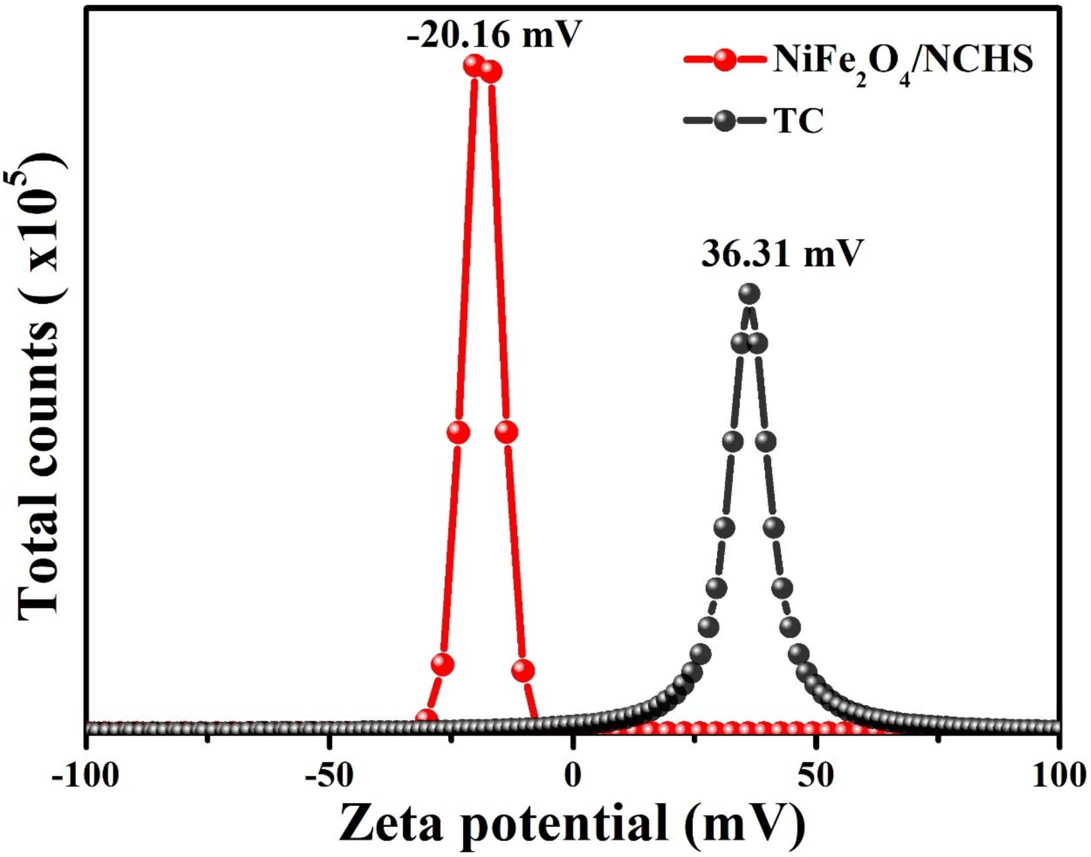

Figure S5. ζ potentials of samples (pH 7)

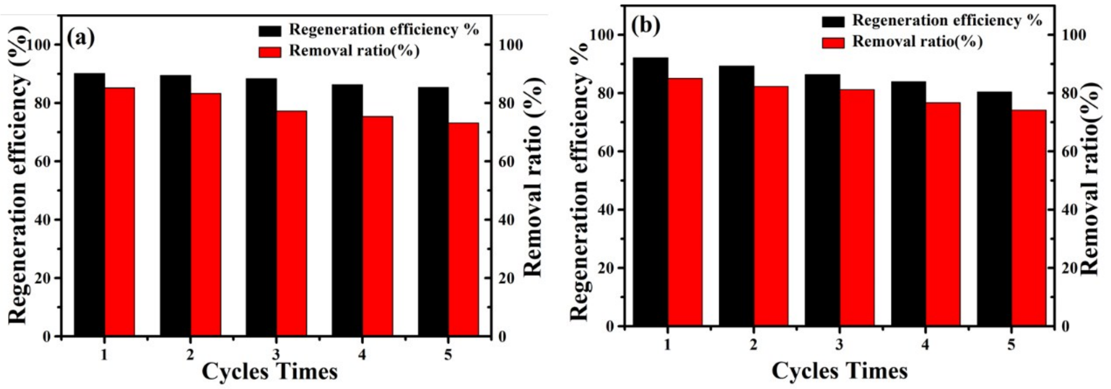

Figure S6. (a) Regeneration efficiency of NaOH (0.001 M) for NiFe2O4/NCHS with tetracycline (b)Regeneration efficiency of NH3·H2O (0.001 M) for NiFe2O4/NCHS with tetracycline (initial tetracycline concentration: 20 mg·L −1, pH: 5, adsorbent dosage: 0.05 g,temperature: 298 K).

■ REFERENCES

(1) L.L. Lian, J.Y. Lv, D.W. Lou, *ACS Sustainable Chem. Eng*. 2017,**5**,10298-10306.

(2) Y. Gao, Y. Li, L. Zhang, H. Huang, J. Hu, S.M. Shah, X. Su, *J. Colloid Interface Sci.* 2012, **368**,540-546.

(3) Y.X. Lin, S. Xu, L. Jia, *Chem. Eng. J.* 2013,**225**,679–685.

(4) S.W. Zhang, H.H. Gao, J.X. Li, Y.S. Huang, A. Alsaedi, T. Hayat, X.J. Xu, X.K. Wang, *J. Hazard.Mater.* 2017,**321**,92–102.

(5) L. Zhang, X.Y. Song, X.Y. Liu, L.J. Yang, F. Pan,J.N. Lv, *Chem.l Eng. J.* 2011,**178** ,26-33.

(6) X. Song, D. Liu, G. Zhang, M. Frigon, X. Meng, K. Li, *Bioresour. Technol*. 2014, **151** ,428−431.
